# Impact of Genetic Variation in Gene Regulatory Sequences: A Population Genomics Perspective

**DOI:** 10.3389/fgene.2021.660899

**Published:** 2021-07-02

**Authors:** Manas Joshi, Adamandia Kapopoulou, Stefan Laurent

**Affiliations:** ^1^Department of Comparative Development and Genetics, Max Planck Institute for Plant Breeding Research, Cologne, Germany; ^2^Institute of Ecology and Evolution, University of Bern, Bern, Switzerland

**Keywords:** regulatory evolution, natural variation, functional non-coding elements, population genomics, selection, tests for selection

## Abstract

The unprecedented rise of high-throughput sequencing and assay technologies has provided a detailed insight into the non-coding sequences and their potential role as gene expression regulators. These regulatory non-coding sequences are also referred to as cis-regulatory elements (CREs). Genetic variants occurring within CREs have been shown to be associated with altered gene expression and phenotypic changes. Such variants are known to occur spontaneously and ultimately get fixed, due to selection and genetic drift, in natural populations and, in some cases, pave the way for speciation. Hence, the study of genetic variation at CREs has improved our overall understanding of the processes of local adaptation and evolution. Recent advances in high-throughput sequencing and better annotations of CREs have enabled the evaluation of the impact of such variation on gene expression, phenotypic alteration and fitness. Here, we review recent research on the evolution of CREs and concentrate on studies that have investigated genetic variation occurring in these regulatory sequences within the context of population genetics.

## Introduction

The initial human genome sequencing project revealed that the proportion of the total genome translated into proteins is ~1.5% (International Human Genome Sequencing Consortium, 2001), while the remaining portion (~98.5%) consists of non-coding DNA. This larger proportion of non-coding DNA is a hallmark of the genomes of higher organisms (Li and Liu, 2019). Evaluating the impact of genetic variation at the coding level is facilitated by a large number of annotated gene models and the simplicity of the genetic code for protein-coding DNA sequences. However, similar studies at the functional non-coding level have suffered from the comparatively sparse annotation as well as the complex and multifarious nature of the regulatory code. In this context, a vigorous debate unfolded as to the amount of functional information carried by the non-coding genome and eventually led to the broad acceptance that while essential, non-coding functional elements amount to a modest proportion of the total non-coding DNA (Doolittle, 2013; Graur et al., 2013; Rands et al., 2014; Huang et al., 2017).

In the last decade, advances in sequencing and assay technologies have contributed to the annotation of a large number of functional non-coding elements. For example, the ENCODE and modENCODE consortia (The modENCODE Consortium et al., 2011; The ENCODE Project Consortium, 2012) used chromatin immunoprecipitation using sequencing (ChIP-seq) and ChIP-on-chip assays to gather a comprehensive catalog of binding sites for a large number of Transcription Factors (TFs) in human, *Drosophila melanogaster*, and *Caenorhabditis elegans* based on genome-wide binding affinity profiles. The availability of such annotation data, along with genomic variation data, has enabled the exploration of non-coding regions for diversity-based signatures of functional constraint. On the other hand, variants occurring in these regions have also contributed to adaptive evolution (Zhen and Andolfatto, 2012). Hence, analyzing the patterns of constraint and variation in CREs contributes to our understanding of between-species phenotypic differences and the process of adaptation.

In this review, we introduce common approaches used to identify regulatory regions. Following this, we will list some of the statistical tools that are used to infer the action of negative and positive selection on non-coding functional regions. Finally, we list studies that presented analyses of selective forces acting at the level of non-coding genomic elements. We have sorted these studies into two sections, the first containing studies that highlight the action of negative (purifying) selection, while the other containing studies that highlight the action of positive selection on non-coding elements (Pollard et al., 2006; Prabhakar et al., 2006; Gittelman et al., 2015).

## Annotating Non-Coding Elements Based on Their Biochemical Signatures

Gene expression regulation is in part controlled by functional non-coding genomic elements. Annotating such elements is important to quantify their exposure to natural selection. Such elements can now be identified based on their biochemical signatures using high-throughput techniques. One of the methods to identify potential regulatory elements is DNase-seq. It allows the identification of regions in the genome at which the chromosome has lost its condensed structure and is therefore susceptible to interactions with available TFs and cleavage by the DNase I nuclease. Such loci are termed DNase I hypersensitive sites (DHSs) and are localized by sequencing the DNA fragments cleaved by the nuclease and mapping them to the reference genome (Sullivan et al., 2015). Another method to assess genome-wide chromatin accessibility is the assay of transposase accessible chromatin using sequencing (ATAC-seq), which is considered faster and more sensitive compared to DNase-seq (Buenrostro et al., 2016). Although loci identified by DNase-seq and ATAC-seq have been shown to be enriched in TF binding sites (TFBS; Calviello et al., 2019), these methods do not provide information about the nature of interacting TFs. On the other hand, ChIP-seq can be used to identify binding sites for a specific TF. In this method, the TF of interest is allowed to bind to its putative binding sites before the DNA is sheared by sonication. TF-DNA bound complexes are then extracted using a TF-specific antibody and DNA is dissociated from the TF and finally sequenced and aligned to the reference genome to identify enriched regions (ChIP-seq peaks; Park, 2009).

## Annotating Non-Coding Elements Using Evolutionary Constraint

The availability of whole-genome sequence data from multiple species has enabled the detection of non-coding genomic regions with extreme sequence conservation at various phylogenetic levels. Conservation at these regions is generally thought to be caused by the presence of functional non-coding elements exposed to similar levels of negative selection across a set of species (Sandelin et al., 2004; De La Calle-Mustienes et al., 2005; Pennacchio et al., 2006). Comparative genomic analysis of conserved elements is therefore an efficient approach to detect non-coding elements involved in the regulation of developmental pathways that are common to many higher organisms. Here we list studies that have attempted to identify such conserved elements using different sets of species.

Visel et al. (2007) used a combination of comparative genome analyses coupled with experimental validations to identify tissue-specific human enhancers. Conserved non-coding elements (CNEs) were identified based on conservation across large evolutionary distances (i.e., non-mammalian vertebrates) and tissue-specificity was established using transgenic mice experiments. Additionally, they also identified ultra-conserved elements (UCNEs), defined as being at least 200 bp long and sharing 100% sequence identity between human, mouse, and rat genomes. This dataset is accessible through the VISTA Enhancer Browser,^1^ which is actively maintained and currently contains 3,148 *in vivo* tested elements. Woolfe et al. (2007) identified CNEs through multiple pairwise alignments of *Fugu* (pufferfish) and four mammalian genomes (human, mouse, rat, and dog), where CNEs are defined as sequences with 65% identity and are at least 40 bp long. They highlighted the association of the identified CNEs with known developmental genes. Lee et al. (2007) determined CNEs that are associated with Transcription Factors in vertebrate genomes, where CNEs from human to mouse were defined as sharing at least 70% identity and being at least 100 bp long, while CNEs from human to *Fugu* had to share at least 65% identity and being at least 50 bp long. The relaxed criteria for human and *Fugu* genome comparison account for the larger evolutionary distance separating the two species. In addition to this, varying proportions (ranging from 0.63 to 10.45%) of human-*Fugu* CNEs were also identified to be overlapping with regions that are experimentally verified TFBS for various genes, indicating the potential role of CNEs in regulating transcription. Persampieri et al. (2008) described ~73,000 CNEs with at least 50% sequence identity between humans and zebrafish and with a length of at least 50 bp. This collection is accessible through the *cne-Viewer*.^2^ Engström et al. (2008) determined highly conserved non-coding elements (HCNEs) across multiple metazoan species using pairwise whole-genome alignments. The threshold of sequence identity used to define an HCNE for each pair of species ranged from 70 to 100%. This dataset is accessible through the ANCORA database.^3^ Dimitrieva and Bucher (2013) highlighted UCNEs by comparing the whole genome sequences of human and chicken, where every UCNE was required to have at least 95% sequence identity and a minimal length of 200 bp. In addition to UCNEs, they also highlight ultra-conserved genomic regulatory blocks (UGRBs), which are clusters of UCNEs that show conserved synteny across different vertebrates. They also annotated a subset of their UCNEs as being putative regulatory elements for developmental genes. This collection of UCNEs and UGRBs is available through the UCNEbase website.^4^ Lomonaco et al. (2014) also determined ultra-conserved elements, where every element had to have 100% sequence identity across human, mouse, and rat, in addition to a minimal length of 200 bp.^5^ Dousse et al. (2016) identified CNEs across five clades of vertebrates, where every CNE was identified using the software *phastCons* (Siepel et al., 2005). This collection of CNEs is available in the CEGA database.^6^ Polychronopoulos et al. (2017) have compiled a list with all publicly available CNE datasets.

## Methods for Detecting Selection

Inferring the action of selective pressure on the non-coding elements (NCEs) has been one of the central challenges for selection-based studies. One of the major limitations for such studies has been sparse annotation data for regulatory regions. Coding elements in the genome tend to be well-annotated, however the same is not true for the non-coding elements. However, this has been partly overcome due to advances in sequencing technologies, like RNA-seq, ChIP-seq, DNase-seq, etc. Comparative genomics studies have used these biochemical signatures to make an informed guess of the potentially functional NCEs. Various metrics have been introduced to detect selective pressures acting on genomic sequences and the fitness consequences of new mutations by using available regulatory annotation. *phastCon*s (Siepel et al., 2005) uses multiple sequence alignment information to identify evolutionarily conserved elements by employing a phylogenetic hidden Markov model. *INSIGHT* (Arbiza et al., 2013) detects the influence of selection on TFBS (ChIP-peaks) based on polymorphism and divergence data; resembling the MacDonald Kreitman (MK) test, named after John H McDonald and Martin Kretiman, who first tested their approach on the *Adh* locus in *D. melanogaster* (McDonald and Kreitman, 1991). The starting point of the MK test is a contingency table summarizing the number of polymorphic (intra-specific) and divergent (inter-specific) variants separately for non-synonymous and synonymous sites. Variants that strongly enhance adaptation tend to fix rapidly in the population and hence contribute less to the polymorphism (within species variation) compared to divergence (between species variation). The MK framework has been used to estimate the proportion of adaptive substitutions that are driven by positive selection within the population of species, a parameter denoted *α*. One of the key shortcomings of this approach is its sensitivity to the presence of slightly deleterious mutations, which can severely bias its estimates (Haller and Messer, 2017). However, INSIGHT overcomes this by using a probabilistic model that explicitly accounts for the presence of weak negative selection. Key quantities estimated by *INSIGHT* are the proportion of selected sites and the number of adaptive substitutions and weakly deleterious variants. *fitCons* (Gulko et al., 2015) clusters unannotated sequences based on their epigenetic markers and uses *ρ* metric (probability of a nucleotide within a functional non-coding element to be under selection) inferred from *INSIGHT* to estimate the probability of a new mutation having a potential fitness effect. *LINSIGHT* (Huang et al., 2017) employs neural networks to make an overall estimate of *ρ* for different genomic features. Here, *ρ* gives an estimate of which feature is most predictive of fitness for any given positional mutation in the genome. *LASSIE* (Huang and Siepel, 2019) accumulates information on all point-specific mutations within non-coding regions and estimates the selection coefficient of every mutation using a maximum likelihood algorithm. One of the central drawbacks of *fitCons* is that the clustering algorithm is dependent on the epigenomic and annotation signatures and is independent of the evolutionary properties. *fitCons2* (Gulko and Siepel, 2019) addresses this by finding clusters of sites that are distinct in evolutionary and epigenomic properties. Kircher et al. (2014) developed a metric, C-score, which predicts the deleterious effect of a new mutation and is comparable across different sites (non-synonymous, synonymous, regulatory, etc.; Racimo and Schraiber, 2014). Finally, a widely used metric to identify elements under selection is the GERP score (Davydov et al., 2010). This score reflects the decrease of substitutions in an inter-species sequence alignment compared to the neutral expectation. Liu and Robinson-Rechavi (2020) proposed a new method to infer the action of selective forces on TFBSs. This method employs Support-Vector Machines, a machine learning approach, to infer the changes in the binding affinity of the TFBS due to variants and does not necessitate a prior definition of “neutral sites.” Here, variants that aid in adaptation would be expected to improve the binding affinity model of the TFBS and will be consequently maintained under positive selection.

## Population Genomics Analyses of Purifying Selection at Non-Coding Functional Elements

Deleterious mutations are usually associated with some detrimental effect on the fitness of the species. These mutations are usually subjected to the force of purifying selection and are either lost or are maintained in lower frequency within the population of species. Given their low frequency, they usually do not contribute to the between-species diversity. Here, we document various studies that have highlighted the action of purifying selection in various species.

Torgerson et al. (2009) analyzed the genetic variation at Conserved non-coding sequences (CNCs) using sequencing data from 35 human samples (20 European Americans and 15 African Americans). CNCs are non-coding sequences that are conserved within a population of species/within a group species. Certain functional studies interpret conservation as a proxy for functionality, hence use conserved elements as potential candidates in their study. For this study, CNCs are defined as non-coding sequences that are conserved in both the human and mouse genome (with at least 70% sequence identity and a minimal length of 100 bp). They report a higher proportion of rare derived alleles in CNCs as compared to synonymous and intergenic sites, indicating the presence of slightly deleterious alleles in CNCs consistent with functional activity. In addition to interpreting summary statistics of genetic variation data, they also reported negative estimates of the population scaled selection coefficient (*γ* = 2Ns) for CNCs in the flanking regions of genes. These observations indicate that the CNCs are under a comparatively higher influence of selective constraints as compared to the intergenic and synonymous sites. Mu et al. (2011) used genomic variation data from the 1000 genomes project to analyze patterns of polymorphism on various aspects of TF-binding sites. They found that ChIP-seq peaks harbored an excess of low-frequency SNPs and structural variants (SVs) as compared to motifs not bound by TFs. Using chimpanzees as the outgroup for the divergence study, they also showed that TF-bound motifs had lower SNP divergence as compared to unbound motifs. In a typical ChIP-seq analysis, post precipitation and sequencing, the reads are mapped to the reference genome, and the areas with the highest coverage are identified as ChIP-seq peaks. These peaks typically contain a consensus binding motif for the protein of interest. The Site Frequency Spectra for polymorphic sites and structural variants showed a significant excess of rare alleles in TF-bound motifs as compared to the broader peak regions. This study also showed that regions associated with TF-binding activity are under higher purifying selection compared to non-functional regions, and that intensity of this selection increases with proximity to coding regions of genes. Vernot et al. (2012) measured nucleotide diversity in DNA binding motifs from 732 TFs that overlapped with DNase I peaks from 138 cell and tissue types, using whole-genome sequencing data of 53 human individuals from five populations available in the Complete Genomics database.^7^ They showed that while diversity varies by over seven-fold across binding motifs (from 2.67 × 10^−4^ to 2.0 × 10^−3^), 60% of binding motifs have lower mean diversity than fourfold degenerate sites, consistent with exposure to purifying selection and hence functional constraint. Their results also highlighted an important heterogeneity in diversity levels between binding motifs, with HOX-, POU-, and FOX-domain factors, which are enriched in controllers of development and cell differentiation, displaying particularly low diversity. Diversity measured in DNase I peaks was significantly lower when peaks were shared by multiple cell types. Similar results were obtained for *Saccharomyces cerevisiae* by Connelly et al. (2013), who quantified the strength of purifying selection acting on binding motifs using genetic variation in 37 strains by employing the metric *Neutrality Index* (*NI*; Rand and Kann, 1996). Out of the 133 binding motifs in their study, 63 had a value of *NI* larger than the one obtained for non-synonymous sites indicating a marked exposure of binding motifs to purifying selection. In this study, the authors also used the *NI* to measure selective constraint at individual intergenic regions. In plants, Haudry et al. (2013) used the whole genome sequence information from nine Brassicaceae species to compare selective constraints acting on CNCs and four-fold degenerate sites using *phastCons* and the folded Site Frequency Spectrum (SFS). SFS is a summary statistic, used extensively in population genetics, which summarizes the distribution of allele frequencies within a population of species. A folded SFS uses the minor allele frequency, i.e., the allele that is the least frequent, to construct the SFS. 90,000 CNCs were identified using *phastCons* analyses on a phylogeny of nine Brassicaceae, representing around 3.8% of the non-coding regions analyzed in this study. In addition to this, they used the population-level data on two of the nine species, namely *A. thaliana* and *C. grandiflora*, to check for a similar signal of conservation within populations. They highlighted that for the population of both species, CNCs displayed an excess of low-frequency minor alleles and lower nucleotide diversity as compared to four-fold degenerate sites, consistent with the action of purifying selection, although this signal was weaker than for the highly conserved zero-fold degenerate sites. De Silva et al. (2014) analyzed patterns of variation at CNCs from CONDOR, a database of developmentally associated CNEs across vertebrates (Woolfe et al., 2007), and obtained multiple alignments for seven vertebrate species (*Homo sapiens*, *Macaca mulatta*, *Mus musculus*, *Gallus gallus*, *Xenopus tropicalis*, *Danio rerio*, and *Takifugu rubripes*). They categorized CNCs into two different classes: non-variable regions (NVRs) which are invariant across all species and restricted variable regions (RVRs) which have at least one variable site across all species (excluding humans). When comparing the SFS of CNCs with synonymous regions (negative control), they observed that CNCs have an excess of rare derived alleles indicating CNCs to be under purifying selection. More specifically, they observed that NVRs have a stronger signal of purifying selection compared to RVRs suggesting that the increased substitution rate observed at RVRs in humans is due to relaxed constraint and not adaptation. They also infer that NVRs harbor a larger proportion of detrimental sites (32%) as compared to that of non-synonymous sites (21%), indicating NVRs to be at a comparatively higher level of purifying selection as compared to non-synonymous regions. Naidoo et al. (2018) used genome-wide polymorphism data from several human populations (Drmanac et al., 2010; Schlebusch et al., 2012; The 1000 Genomes Project Consortium, 2015) and non-coding annotation from the ENCODE project (The ENCODE Project Consortium, 2012). They calculated nucleotide diversity and Tajima’s D as a statistical measure of constraints acting on different classes of genome sequences. Tajima’s D is a summary statistic that compares the average number of pairwise differences with the average number of segregating sites within a population. Negative values of Tajima’s D indicate an excess of rare alleles and can be used to identify regions where genetic variants segregate at lower frequencies than at neutral loci, a signal consistent with the action of negative selection. They observed coding regions to experience the highest level of purifying selection, closely followed by promoters and untranslated regions (UTRs), while enhancers were the least constrained. This study highlights that on average, regulatory elements in proximity to the coding regions displayed stronger purifying selection as compared to distal ones.

To summarize, the level of constraints acting on CNCs seems to be intermediate when compared with other coding and non-coding sequences. Overall, CNCs are reported to be under higher constraints when compared with synonymous sites and other intergenic regions, and under lower constraints when compared with non-synonymous sites. Such observations could indicate that CNCs are composed of different combinations of binding affinity and non-binding affinity motifs. Hence, the intermediate levels of constraints cannot be directly translated to an overall intermediate level of selection intensities.

## Population Genomic Analyses of Positive Selection at Non-Coding Functional Elements

Beneficial mutations represent a small fraction of all naturally occurring mutations, but they are important for adaptation to varying environmental conditions. Given their positive contribution to fitness, such mutations tend to rapidly increase in frequency, and eventually fix, within populations and species. When the number of beneficial mutations responsible for a new adaptive phenotype is small, their rapid increase in frequency generates a characteristic signature in polymorphism data referred to as a selective sweep (Cutter and Payseur, 2013). Several studies have documented selective sweeps in non-coding regions and demonstrated how the beneficial allele modified the expression of the target gene.

Schlenke and Begun (2004) studied the within-species diversity in North-American and African populations of *Drosophila simulans* in the 2R chromosome, a freely recombining region of the genome. The levels of heterozygosity in the 100 kb region under study were reported to be significantly reduced specifically in the North-American population; potentially indicating a recent selective sweep. In this genomic region, they identified a fixed insertion of a transposon in the 5' end of the *Cyp6g1* gene in the American population, correlated with increased transcript abundance, and which had been previously associated with insecticide resistance in *D. melanogaster*. Chan et al. (2010) studied the loss of pelvis phenotype in certain pelvic-reduced stickleback fish populations by performing F1 crosses between pelvic-complete and pelvic-reduced fishes in an experimental setting. They highlighted the loss of an enhancer, *Pel*, for the *Pitx1* gene (expressed in hindlimbs of many vertebrates) in pelvic-reduced fishes to be the driver for the loss of the pelvis. They showed that the heterozygosity at the *Pel* enhancer is significantly less in the pelvic-reduced compared to pelvic-complete populations, could not be explained solely by population size bottlenecks, and is therefore consistent with the expected signature of a selective sweep. *LCT*, the gene coding for the lactase enzyme, is a well-described example for recent selective sweeps in humans (Enattah et al., 2002; Bersaglieri et al., 2004; Tishkoff et al., 2007). The geographical distribution of the persistence of this enzyme into adulthood is shown to be associated with dairy farming (Enattah et al., 2002), hence the ability to digest lactose during adulthood varies in different populations. This lactase persistence has been proposed to be regulated by cis-acting elements (Wang et al., 1995). Enattah et al. (2002) highlighted two alleles, that are located within the intronic regions of the *MCM6* gene, in the Northern European population that are associated with lactase persistence into adulthood. Bersaglieri et al. (2004) further highlighted the high between-population differences in the frequency of these persistence markers.

Generally, beneficial mutations are rare compared to deleterious mutations, and detecting them is difficult for multiple reasons: confounding demographic parameters can leave similar signatures in the genome, the selective sweep may be too old and the beneficial allele fixed within the population or, in the case of a polygenic adaptation model, the signal for individual loci under positive selection is too weak to be detected (Barghi et al., 2020). Hence, highlighting the effect of positive selection on a single locus is challenging. In addition to this, the effects of underlying background selection are important influencing factors in studies highlighting genome-wide scans of positive selection (see Charlesworth and Jensen, 2021). Some population genomics studies tried to overcome this problem by aggregating the signal carried by genetic variation over multiple loci.

Kudaravalli et al. (2009) tested whether SNPs with a significant association with gene expression (eQTLs) are frequent targets of selection in humans. For this, they analyzed HapMap (International HapMap Consortium, 2003) genomes from three different human populations: Asian, Central European, and Yoruban together with gene expression data from lymphoblastoid cells obtained for all 210 unrelated HapMap individuals. To detect the signature of recent or ongoing positive selection on eQTLs they used the iHS (integrated haplotype score; Voight et al., 2006), a powerful approach to detect selection when the beneficial mutation has not been fixed and is segregating at a frequency between 50 and 85%. Their results showed that SNPs surrounded by signatures of positive selection were more likely to be eQTLs compared to random SNPs, leading to the suggestion that selection on transcript levels is an important aspect of human adaptation. More broadly, this study also showed how logistic regression models can serve as an appropriate statistical approach to test for associations between signals of positive selection and molecular phenotypes. Haddrill et al. (2008) analyzed polymorphism and divergence at 67 coding and non-coding elements in *D. simulans* (*n* = 20). They observed excess of low-frequency alleles in the SFS for introns, 5' UTRs, and 3' UTRs, indicative of selective constraints in those non-coding regions. Based on a MacDonald-Kreitman (MK) analysis, they also reported that the proportion of adaptive substitution, α, for 5' UTRs and 3' UTRs is comparable with non-synonymous sites in this species. This study highlights that UTRs in *D. simulans* have been under both positive as well as negative selection.

Torgerson et al. (2009) analyzed genetic variation at CNCs using resequencing data from 35 human samples (20 European-Americans and 15 African Americans) using the chimpanzee genome as the outgroup. In this study, CNCs were defined as non-coding sequences conserved in human and mouse. They discretized genomic data into GO functional categories to identify GO categories that are significantly associated with selection in CNCs. For this purpose, they use a modified version of the MK test, *mkprf* (Bustamante et al., 2002), which infers population scaled selection coefficient at individual loci (*γ*). In African-Americans, three categories associated with positive selection in CNCs were regulation of cellular processes, protein modification, and cell cycle, while categories associated with negative selection were cytosol, ribosome, extracellular region, and carrier activity [with false discovery rate (FDR) < 25%]. In European-Americans, the categories associated with positive selection were calcium ion binding, organelle organization and biogenesis, cell cycle, and behavior (with FDR < 25%), while categories associated with negative selection were proteinaceous extracellular matrix and extracellular space (with FDR < 20%). By analyzing selection pressures acting on genomic data grouped into the functional categories, this study highlights population-specific functional categories that are more likely to be targets of selective forces. He et al. (2011) analyzed TFBS in *D. melanogaster* and *D. simulans* for 30 TFs from REDfly (Rivera et al., 2019), a curated collection of known insect *cis*-regulatory modules. Using a Position Weight Matrix scoring approach, they predicted the effect of each SNP on TFBS binding affinity and measured the frequencies of TFBS-modifying alleles. The unfolded SFS of affinity-decreasing mutations is skewed towards low frequency derived alleles suggesting that negative selection acts to maintain existing TFBS. Furthermore, the results of MacDonald-Kreitman analyses on both affinity increasing and decreasing mutations indicated that positive selection enabled gains and losses of TFBS in both species. Vernot et al. (2012), in addition to evaluating the selective constraint acting on DHSs (see the previous section), used the same dataset to conduct a genome-wide scan for signatures of positive selection at the level of regulatory regions. For this, they used the LSBL metric (Shriver et al., 2004) to identify DNase I peak with significant allele frequency differences across human populations compared to the genomic background. DHS peaks falling in the top 1% of the genome-wide distribution of LSBL values were considered potential targets of positive selection and genes located within 50 kb of those candidate DHS peaks were tested for enrichment of KEGG pathways.^8^ Impressively, among the 15 enriched pathways, the authors identified the melanogenesis pathways in the European population only, suggesting that in addition to already described coding-variants, regulatory changes contribute, as well, to the evolution of adaptive pigmentation phenotypes in this population. Interestingly, the authors also show that the proportion of highly differentiated DHS differs across the four cell types surveyed in their study.

Arbiza et al. (2013) analyzed polymorphism and divergence data at binding sites with ChIP-seq peaks from the ENCODE project using genomic data from 54 unrelated human individuals and three primate genomes. Based on their new probabilistic implementation of the MK framework (INSIGHT), they estimate the proportion of selected sites in binding sites within ChIP peaks to be 0.33 (*vs.* 0.80 for second codon positions). They also identify a large variation in the proportion of selected sites across TFs that is largely explained by differences in the information content of the associated binding models. Binding sites also carried a significant signal of positive selection, such that 1 out of 8,300 nucleotides in TFBS was estimated to have been fixed through positive selection and 1 out of 20 recently fixed alleles are adaptive substitutions. Among all TF analyzed in this study, binding sites for the Zinc finger TF GATA2 and GATA3 displayed the largest number of adaptive substitutions (312). Huang et al. (2017) studied cell and tissue-specific constraints acting on enhancers. They obtained a comprehensive enhancer annotation list in humans from a study by Andersson et al. (2014). They introduced LINSIGHT, a method to estimate the fitness consequence of mutation in non-coding regions. They showed that enhancers in tissues associated with sensory perception, the immune system, and the male reproductive system have low LINSIGHT scores, suggesting that these enhancers are under low constraints and could potentially be under positive selection. They also point out that enhancers active in tissues associated with the female reproductive system are under higher constraints as compared to tissues associated with the male reproductive system. Along with the introduction of LINSIGHT, this study highlighted that the fitness consequence of mutations in enhancers is dependent on many aspects, including cell and tissue specificity, and constraints acting on the promoters of the target gene.

## Discussion

Concerning purifying selection, polymorphism- and divergence-based studies highlighted in this review (to exemplify — Torgerson et al., 2009; De Silva et al., 2014) demonstrate that certain non-coding elements in the genome seem to be under constraint, indicating the action of purifying selection. Interestingly, De Silva et al. (2014) pointed out that highly conserved CNCs across various vertebrates appear to be under higher levels of constraints as compared to non-synonymous sites. DNA binding motifs within corresponding ChIP-seq peaks, one of the most precise annotations of functional non-coding elements across various species, have been shown to be under higher levels of purifying selection as compared to fourfold degenerate sites in humans (Vernot et al., 2012), and, as compared to non-synonymous sites in yeast (Connelly et al., 2013). However, overall, functional non-coding classes show varying patterns of purifying selection which is intermediate to synonymous and non-synonymous sites (Haddrill et al., 2008; Torgerson et al., 2009). Often regulatory modules have been highlighted to be under higher constraints as compared to other non-annotated and non-coding regions, as an example TFBS motifs are under higher constraints as compared to unbound motifs (Mu et al., 2011). Finally, the intensity of these constraints seems to be higher for elements that are in the proximity of the coding regions as compared to the distal elements (Mu et al., 2011; Naidoo et al., 2018).

Demonstrating the effect of positive selection on individual loci is challenging, hence several studies took the approach of pooling loci-based common non-coding functional annotation and compared the aggregated signal to a neutral reference class. For example, Torgerson et al. (2009) and Vernot et al. (2012) clustered regulatory regions associated with genes based on their functional GO terms and biological pathways that they participate in, respectively, for various human populations. Here, Vernot et al. (2012) highlighted that the pigmentation pathway seems to constitute genes whose regulatory elements could have potentially been targeted by positive selection in the European population, suggesting that regulatory changes could be responsible for adaptive phenotypic changes. Huang et al. (2017) employ their method LINSIGHT to aggregate signals of selection from different tissue types in humans. In some cases, regulatory elements could be under the influence of both positive as well as negative selection, where negative selection maintains regulatory elements and positive selection is responsible for their gain and loss within species as has been pointed out in the studies by Haddrill et al. (2008) and He et al. (2011). Additionally, in some cases, variations in CREs have also been proposed to be paving way for speciation. To exemplify, Mack and Nachman (2017) highlighted that the accumulation of variations within CREs could be linked to post-zygotic isolation which eventually leads to a reduction in inter-species gene flow and, potentially, speciation. Post-zygotic isolation is one of the mechanisms of reproductive isolation where the inter-species hybrid is either inviable or sterile, thus leading to an increase in the inter-species differences. Along similar lines, in a speciation study on *Mus musculus domesticus* and *Mus musculus musculus*, Mack et al. (2016) highlighted the potential role of the accumulation of changes within regulatory elements in speciation.

## Challenges and Pitfalls

With the advent of whole-genome sequencing and assay technologies, the availability of genomic data has grown exponentially. However, one of the central questions remains open, which is — What proportion of the genome is “functional”? In the case of non-coding elements, many studies have highlighted the approach of linking conservation to functionality, however, this could potentially dilute the signature of natural selection (Arbiza et al., 2013). To make this search more precise, a proposed alternative has been to exclusively consider non-coding sequences that display some biochemical signatures. Many comparative genomics studies use these biochemical signatures as starting points to detect patterns of constraints on elements (displaying the signatures) as compared to putative neutral elements and infer functionality and forces of selection in action. Here, constraints and the presence of a biochemical signature, both, act as a proxy for functionality. Along similar lines, in the case of humans, some studies have hypothesized the proportion of functional sites in the genome to be around 4–8% (Ward and Kellis, 2012; Rands et al., 2014), these estimates are based on the proportion of sites that are under constraint. However, changes in the non-constraint regions could also have functional consequences (Ludwig et al., 2000; Dermitzakis and Clark, 2002). Estimating the proportion of genetic variants, within CREs, that contribute to adaptive evolution is challenging, mainly due to a lack of a robust model of neutral versus adaptive evolution, specifically for regulatory regions (Liu and Robinson-Rechavi, 2020).

As compared to coding regions, functional studies are challenging within NCEs due to sparse annotation data. This has been partially overcome with biochemical assays and large-scale annotation projects like ENCODE (humans; The ENCODE Project Consortium, 2012) and modENCODE (*D. melanogaster*; The modENCODE Consortium et al., 2011). However, these assays generally highlight biochemically active regions, which is not a direct indication of functionality (Doolittle, 2013; Graur et al., 2013; Huang et al., 2017). This advocates for the need for refined functional annotations of the non-coding elements. One of the other challenges in functional studies has been to choose appropriate neutral sites, which are sites indifferent to variations. Comparing such sites against “test” sites aid in elucidating the signal of selective forces. However, as highlighted by Casillas et al. (2007), the choice of the genomic class used as neutral reference can lead to under-or over-estimations of the action of selective forces on “test” sites. In addition to selecting neutral regions, the neutral forces associated with the demographic history of the populations should also be factored in to make an informed estimate of the action of selective forces (Zhen and Andolfatto, 2012).

Such methods usually aggregate the signal of selection over multiple loci, as the signal from a single locus is sparse, estimating the marginal contribution of individual loci is difficult (Andolfatto, 2005). To exemplify, in the case of local adaptation, a certain group of loci that contribute to adaptation will be under the action of positive selection and evolve rapidly as compared to the other non-functional sequences. Methods attempting to detect selective pressure will highlight these loci. To aid in interpretation, some studies (Haygood et al., 2007; Torgerson et al., 2009) use Gene Ontology (GO) identifiers to highlight biological categories, and consequently the participating genes, which are likely subject to selection. However, as highlighted by Galtier and Duret (2007), one of the explanations for rapidly evolving elements, besides the action of selection, could also be other factors, such as biased gene conversion, making the inference of selection challenging.

Effective partitioning of the regulatory elements is one of the central challenges for performing functional studies of the non-coding elements. He et al. (2011) highlight an interesting approach of partitioning the regulatory regions into affinity increasing and affinity decreasing sites, similar to synonymous and non-synonymous sites in the coding regions. However, such partitioning is only possible for regulatory elements that have a well-characterized binding model. The new sequencing methods and the rapid rise in sequencing data will help to fine-tune the NCE annotation and establish TF-specific binding models. In addition to this, low-affinity binding sites in NCEs have been reported to play a key role in regulatory robustness by enabling the regulatory elements to harbor multiple binding sites (Crocker et al., 2015; Hajheidari et al., 2019). However, reliable detection of such elements through ChIP-seq experiments has been challenging due to their sparse signal, making them difficult to distinguish from the genomic background noise (Crocker et al., 2015). Enabling reliable detection of such elements will be one of the major challenges for future developments in assay technologies and bioinformatics pipelines.

## Author Contributions

MJ and SL designed the structure of the review. MJ, AK, and SL wrote the review. All authors contributed to the article and approved the submitted version.

### Conflict of Interest

The authors declare that the research was conducted in the absence of any commercial or financial relationships that could be construed as a potential conflict of interest.

## Footnotes

^1^https://enhancer.lbl.gov/

^2^http://bioinformatics.bc.edu/chuanglab/cneViewer/

^3^http://ancora.genereg.net

^4^https://ccg.epfl.ch/UCNEbase/

^5^http://ucbase.unimore.it

^6^http://cega.ezlab.org

^7^https://www.completegenomics.com/public-data/

^8^https://www.genome.jp/kegg/pathway.html
